# Application of GFAT as a Novel Selection Marker to Mediate Gene Expression

**DOI:** 10.1371/journal.pone.0017082

**Published:** 2011-02-14

**Authors:** Guogan Wu, Yu Sun, Wei Qu, Ying Huang, Ling Lu, Lun Li, Weilan Shao

**Affiliations:** 1 Research Center for Biotechnology and Biomass Energy, Nanjing Normal University, Nanjing, People's Republic of China; 2 Jiangsu Key Laboratory for Microbes and Functional Genomics and College of Life Sciences, Nanjing Normal University, Nanjing, People's Republic of China; 3 Faculty of Veterinary Science, University of Sydney, Camden, New South Wales, Australia; University of Kent, United Kingdom

## Abstract

The enzyme glutamine: fructose-6-phosphate aminotransferase (GFAT), also known as glucosamine synthase (GlmS), catalyzes the formation of glucosamine-6-phosphate from fructose-6-phosphate and is the first and rate-limiting enzyme of the hexosamine biosynthetic pathway. For the first time, the GFAT gene was proven to possess a function as an effective selection marker for genetically modified (GM) microorganisms. This was shown by construction and analysis of two GFAT deficient strains, *E. coli Δglm*S and *S. pombe Δgfa1*, and the ability of the GFAT encoding gene to mediate plasmid selection. The *gfa1* gene of the fission yeast *Schizosaccharomyces pombe* was deleted by *KanMX6*-mediated gene disruption and the Cre-loxP marker removal system, and the *glm*S gene of *Escherichia coli* was deleted by using λ-Red mediated recombinase system. Both *E. coli Δglm*S and *S. pombe Δgfa1* could not grow normally in the media without addition of glucosamine. However, the deficiency was complemented by transforming the plasmids that expressed GFAT genes. The xylanase encoding gene, *xynA2* from *Thermomyces lanuginosus* was successfully expressed and secreted by using GFAT as selection marker in *S. pombe*. Optimal glucosamine concentration for *E. coli Δglm*S and *S. pombe Δgfa1* growth was determined respectively. These findings provide an effective technique for the construction of GM bacteria without an antibiotic resistant marker, and the construction of GM yeasts to be applied to complex media.

## Introduction

Genetic engineering has been the most revolutionary technology in the past decades, and has had profound impacts on the genetic modification of microorganisms for food production, biopharmaceutical drugs, vaccine development and environmental remediation [1]. Establishing suitable selection marker genes (SMGs) has been one of the hot topics in the field because it is essential for the identification and selection of cells transformed by heterogeneous gene(s), and it is also frequently required for maintaining the modified property in the growing cells of genetically modified (GM) microbes.

Current SMGs that have been widely used in microbial engineering are antibiotic SMGs and auxotrophic SMGs. In genetic engineering of bacteria such as *Escherichia coli*, *Bacillus* spp. and *Lactobacillus* spp., the selection and maintenance of recombinant cells is highly dependent on antibiotic SMGs. These SMGs have the advantage of being convenient and efficient in the construction of recombinant strains in laboratory and the production of recombinant proteins in fermentors. Auxotrophic SMGs have been successfully used in genetic engineering of yeasts and filamentous fungi, e.g. the genes such as *ura4*, *his3*, *his7*, *CAN1*, and *lys2* have been successfully applied to the auxotrophic strains of fission yeast *Schizosaccharomyces pombe* [2]–[6]. Under laboratory conditions, when a single gene related to the biosynthesis of an amino acid or nucleotide is defected, the auxotrophic cells cannot grow on a selective medium containing no corresponding compound, unless they are transformed by recombinant DNA to supplement the deficient gene.

As the rapid growing of application of GM microorganisms in a broad area of biotechnology, the development of bio-safe and efficient SMGs has become a major mission for researchers in genetic engineering [7], [8]. There has been concern that antibiotic SMGs can spread antibiotic resistance to pathogenic microbes and pose a threat to environment and human/animal health when the GM microbes carrying such SMGs are applied to the area other than laboratory and manufacturer's fermentors [2]. Therefore, there is a demand for bio-safe SMGs for GM microbes in the areas of food fermentation, health-promoting microflora, livestock additives, and environmental remediation [2], [3]. Another important concern for current SMGs is their selection ability in various micro environments. In reality, the GM microbes carrying antibiotic SMGs will lose the modified property shortly after they are grown in the media without the addition of antibiotic drugs. For auxotrophic SMGs, the selection medium should not contain any trace substance that can be synthesized by the SMG, otherwise the medium will lose its selectiveness to the recombinant cells. This selective condition cannot be mimicked inside the digestive system of human or animals or in the fermentation broth containing bio-materials which are the mixture of amino acids, sugars and nucleotides. Therefore, it is important to develop alternative SMGs with features of both biosafety and effective selectivity under natural conditions.

Glutamine: fructose-6-phosphate aminotransferase (GFAT, EC 2.6.1.16), also known as glucosamine-6-phosphate synthase (GlmS), catalyzes the formation of glucosamine-6-phosphate (Glc-6-P) using glutamine as the ammonia donor. GFAT is the first and rate-limiting enzyme of the hexosamine biosynthetic pathway (HBP) that controls the availability of precursors for amino sugar containing macromolecules [9]. The gene encoding GFAT has been cloned and analyzed in a number of microorganisms, such as *Escherichia coli* [10], *Candida albicans* [11], *Saccharomyces cerevisiae* [12], *Aspergillus niger* [13], and *Volvariella volvacea* (edible straw mushroom) [14]. GFAT genes have been designated as *gfa1*, *gfaA*, *gfat*, and *glm*S in *S. pombe*, *A. niger*, *V. volvacea* and *E. coli*, respectively. It has been shown that a disruption of GFAT gene is vital in some species, however, those mutant cells can resume growth when glucosamine is added to the media [11]-[13], [15]. Therefore, *gfaA* was suggested as a potential SMG by Arthur *et al.* in their work on *A. niger* [13]. However, the practical application of *gfaA* as an SMG has not been reported for any strain of microorganisms up to date.

Presently, *E. coli* is an extensively used GRAS (Generally Recognized As Safe) model microorganism in scientific research and industrial production of recombinant proteins, and *S. pombe* is an excellent model of eukaryotic cells for studying different questions in cell biology. The work presented here was designed to construct two GFAT deficient strains of *E. coli* and *S. pombe*, designated as *E. coli Δglm*S and *S. pombe Δgfa1*. The deficient strains were used to assess the potential of the GFAT gene as a novel SMG. The culture conditions of both strains were also tested to provide practical solutions, which included the determination of behavior in different media systems, their sensitivity to the components of the medium (especially glucosamine) and the ability to maintain the selection stress that supports the expression of a foreign gene. The results reported in this paper offer a useful resource for the construction of GM microbes for various applications.

## Materials and Methods

### Strains and media

*E. coli* strain DH10B was used as the host for gene cloning and construction of recombinant plasmids, and strain K12 was employed for the construction of *glm*S deficient host. *E. coli* cells were routinely grown aerobically in Luria-Bertani (LB) medium at 37°C, and ampicillin was added at a final concentration of 100 mg/l when necessary. Unless described, all chemicals were purchased from Sigma–Aldrich (Missouri, USA).

The *S. pombe* strain YHL6381 (h^+^, *his3*-D1, *leu1*-32, *ura4*-D18, *ade6*-M210) was used in this study. Yeast extract supplements (YES) medium and Edinburgh's minimal medium (EMM) were used as described by Alfa *et al.* [16]. Thiamine, geneticin (G418) and glucosamine were added at 5 µM, 100 mg/l and 10 mM respectively when necessary. Growth of cells was monitored by measuring the optical density at 600 nm (OD_600_).

### Genes, plasmids, and DNA manipulation

The *V. volvacea* cDNA library, constructed by our lab previously, was used as the template for amplifying the *gfat* cDNA fragment. The codon-optimized xylanase gene from *Thermomyces lanuginosus* DSM 5826, *xynA2*, was obtained by PCR using the recombinant plasmid pHsh-xynA2 as template [17]. Plasmids pFA6a-kanMX6 and pREP3X were used for integration and expression respectively in yeast. The apramycin resistance cassette *aac(3) IV* was obtained by PCR using plasmid pIJ773 as template. The vector pKD46 was used for expression of the three λ-Red recombinases and pCP20 was used for expression of the FLP recombinase in gene disruption in *E. coli* [18]. The novel expression vector pHsh, constructed by our lab [19]–[20], was used for expression of *glmS* in *E. coli*.

DNA isolation, amplification, digestion and ligation were performed by following standard procedures [21] and manufacturers' instructions. Plasmid DNA and PCR products were purified using the Qiagen plasmid kit and PCR purification kit (Qiagen, USA). DNA restriction and modification enzymes were purchased from TaKaRa (PR China). Genomic DNA of *S. pombe* was isolated according to the protocols described by Hoffman and Winston [22]. *E. coli* cells were transformed by electroporation using GenePulser (Bio-Rad, USA), yeast cells were transformed using lithium acetate method [23]. The other procedures of gene manipulation were performed following the standard protocols described by Sambrook *et al.* [21].

### Deletion of the GFAT encoding gene

#### Knockout of the *gfa1* gene in *S. pombe*

**Figure 1:**
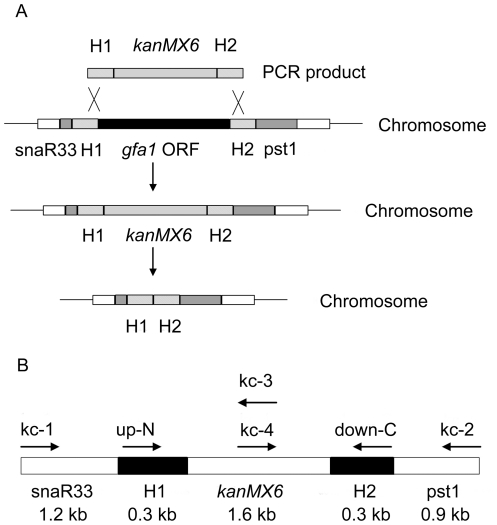
Knockout of the *gfa1* gene and primers used for verification. (**A**) The deletion of *gfa1* gene in the chromosome using *kanMX6*–mediated gene integration cassette in *S. pombe*. H1: the homologous region upstream of the *gfa1* gene, H2: the homologous region downstream of the *gfa1* gene. snaR33, pst1: the genes upstream and downsteam of the *gfa1* ORF. (**B**) The position of the primers used in the deletion verification.

The deletion of *gfa1* gene was performed by homologous recombination [24]. The upstream and downstream flanking sequences of *S. pombe gfa1* (SPBC12C2.11, GenBank accession no. AL031536) were designed as homologous recombination regions. The 300 bp upstream fragment (H1) and downstream fragment (H2) were amplified from the genomic DNA by using 2 pairs of primers: up-N, up-C, and down-N, down-C (Table 1). H1 was cloned into pFA6a-kanMX6 at *Bam*HI and *Bgl*II sites, and H2 was cloned into the same plasmid at *Pme*I and *Eco*RI sites, resulting in the plasmid pFA6aH1-kanMX6-H2. The linear H1-kanMX6-H2 cassette was amplified from pFA6aH1-kanMX6-H2 by using the primers up-N and down-C (Fig. 1A), and transformed into the *S. pombe* cells. The transformants were selected on the solid plate and further cultured in the liquid medium of YES supplemented with G418 and glucosamine. The genomic DNA of G418-resistant transformants was isolated for verifying the integration by PCR using pairs of primers (Table 1): Sp-N and Sp-C, up-N and down-C, kc-1 and kc-2, and kc-3 and kc-4, respectively. Primers kc-1 and kc-2 bound to the regions 1.5 kb and 1.2 kb upstream and downstream of *gfa1* in the chromosome, kc-3 and kc-4 primers were at the region of 0.6 kb downstream of the N terminus of *KanMX*6 cassette (Fig. 1B). The integrated antibiotic *KanMX6* cassette was then excised from the genome using the Cre-loxP-mediated marker removal system to generate *S. pombe Δgfa1* [25].

**Table 1:** Primers used in this study.

Primer code	primer sequence (5′-3′)
up-N	CCCGGATCCTTCAATAGAATTTTTGCAACCGT
up-C	CCCAGATCTTGATAAGTAAAATCGACTTTTCC
down-N	CCCGTTTAAACATTTGATTATCCCTCATTGT
down-C	CCCGAATTCACCATCATTAGTTTCCAAG
Sp-N	CCCGTCGACATGTGGTAAGCTATTTGTTTG
Sp-C	CCCCCCGGGTTATTCCACCGTAACTGATTT
Vv-N	CCCCTCGAGATGTGTGGGATTTTTGCTTACTG
Vv-C	CCCCCCGGGTTACTCCGTAGTGACAGATTTAGC
kc-1	GGCCTGAGTAAATAAAGG
kc-2	CCAATGGTAAGTTCACCA
kc-3	ACCTTTGCCATGTTTCAG
kc-4	CTGAAACATGGCAAAGGT
Ec-N	TTACGTCGTCTGGAATACCGCGGATATGACTCTG CCGGTCTGGCTGTAGGCTGGAGCTG
Ec-C	TCGGTGCCTTTGATCAGCGCGACATGGTAAGC CAGCTGCAGGGGGATCCGTCGACC AGCCAGCTGCAGGGGGATCCGTCGACC
apra-N	TGTAGGCTGGAGCTGCTTC
apra-C	ATTCCGGGGATCCGTCGACC
Ev-N	ATGTGTGGAATTGTTGG
Ev-C	TTACTCAACCGTAACCG
pHsh-N	ACTCTTCCTTTTTCAATATT
pHsh-C	CCCGGTACCCTGTCAGACCAAGTTTAC
Eg-N	ATGTGTGGAATTGTTGGC
Eg-C	CCCGGTACCTTACTCAACCGTAACCGA

#### Deletion of the *glm*S gene in *E. coli*

The λ-Red mediated recombinase system and FLP-mediated excision of the disruption cassette were employed to disrupt the target gene and remove the antibiotic resistant gene, respectively. A DNA fragment including 88 bp of homologous regions of the *glm*S gene of *E. coli* (GenBank accession no. NC000913), FRT site recognized by FLP-recombinase, and the apramycin resistant gene (*apr* ^R^) was obtained by PCR using plasmid pIJ773 as template and Ec-N, Ec-C as primers (Table 1). Subsequent experiment procedures were performed according to the protocols described in [18]. Primers apra-N, apra-C and Ev-N, Ev-C (Table 1) were used for the verification of gene deletion, where Ev-N and Ev-C annealed to the 5′ and 3′ end of the *glm*S of *E. coli*. The *glm*S deficient strain was designated as *E. coli Δglm*S.

### Supplements to media for the deficient strains

#### Determination of glucosamine concentration for *S. pombe Δgfa1*

To examine the growth ability of *S. pombe Δgfa1* in regular media, the cells were streaked on EMM and YES solid media with or without glucosamine. The effect of glucosamine concentration on the growth of *S. pombe Δgfa1* was determined by adding glucosamine to EMM or YES liquid media at different concentrations: 0, 0.25, 0.5, 1, 2, 4, 6, and 8 mM, respectively. *S. pombe* YHL6381 was used as control.

#### Growth condition and medium designed for *E. coli Δglm*S

The optimal requirement of glucosamine for the growth of *E. coli Δglm*S was determined by adding 0, 0.125, 0.25, 0.5, and 1 mM glucosamine into LB medium. Yeast extract or tryptone was added into M9 minimal medium separately to examine their effects on the selectivity of M9 minimal medium over *E. coli Δglm*S. To support a quick selective growth of *E. coli Δglm*S, an undefined medium was designed on the basis of M9 minimal medium by supplementing tryptone and vitamin, and subtracting Mg^2+^ and glucose. The vitamin mixture was prepared as described [26].

### Complementation of the gene deletion from plasmids

#### Complementation of *gfa1* deletion in *S. pombe*

The *LEU2* marker gene was excised from pREP3X by digestion to form the plasmid pREP3Xdl. The 2361 bp *gfa1* gene of *S. pombe* was amplified using the primers Sp-N and Sp-C, digested with *Sal*I, *Sma*I, and cloned into pREP3Xdl to produce the recombinant plasmid pREP3X-gfa1. A 2094 bp *gfat* cDNA of *V. volvacea* was amplified using the primers Vv*-*N and Vv-C, digested with *Sma*I and cloned into pREP3Xdl, resulting in the recombinant plasmid pREP3X-gfat.

The expression plasmids were transformed into *S. pombe Δgfa1* and the transformed cells were plated on EMM and YES medium without glucosamine, respectively. Plasmid pREP3Xdl was used as control in the complementation tests.

#### Complementation of *glm*S deficiency in *E. coli*

The ampicillin resistant gene was excised from the plasmid pHsh by reverse PCR using the primers pHsh-N and pHsh-C with an additional *Kpn*I site, resulting in a linear fragment dpHsh. A 1830 bp *glm*S DNA fragment of *E. coli* was amplified using the primers Eg*-*N and Eg-C (Table 1), digested with *Kpn*I and ligated to dpHsh to generate the recombinant plasmid pHsh-glmS. The plasmid pHsh-glmS was transformed into *E. coli Δglm*S. The transformed cells were added with MT (M9 minimal medium and 1% tryptone) as restoration medium immediately after electroporation and were plated on MT agar medium. Several colonies of the cells were streaked into MT liquid medium and the plasmid was isolated from the culture and verified by single enzyme digestion using *Kpn*I and double enzyme digestion using *Kpn*I and *Hind*III. The plasmid pHsh without *glm*S gene was used as control.

### Heterologous gene expression mediated by *gfat* marker

**Figure 2:**
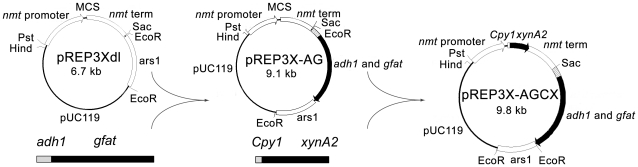
The sketch map of the constructed vector pREP-AGCX.

The expression plasmid pREP-AG was constructed by ligating the *gfat* cDNA of *V. volvacea* along with the *adh1* promoter of *S. pombe* to pREP3Xdl at the *Sac*I site. The xylanase gene *xynA2* amplified from pHsh-xynA2 was cloned into pREP3X-AG at the multiple cloning site to generate plasmid pREP3X-AGX. The signal peptide (SP) of Cpy1 (carboxypeptidase Y, a vacuolar protease) was employed to direct the secretion of target protein [27]. The SP-encoding DNA was fused with the 5′ end of *xynA2* gene in pHsh-xynA2 by inverse-PCR, and the fused gene, SP-*xynA2*, was amplified and cloned into pREP-AG to generate plasmid pREP-AGCX (Fig. 2). As a control, plasmid pREP3Xdl-xynA was constructed by cloning SP-*xynA2* into the vector pREP3Xdl.

Plamids pREP-AGCX, pREP-AGX and pREP3Xdl-xynA were transformed into *S. pombe Δgfa1* and the transformed cells were plated on EMM medium supplemented with thiamine. Colonies were streaked into 5 ml liquid EMM medium without adding thiamine to derepress the *nmt1* promoter to allow the expression of *xynA2*. The cells were cultured in the medium for 24 h at 32°C, and harvested by centrifugation at 8,000 *g* for 5 min. The supernatants were kept on ice. While pipetting 20 µl out for the assay of xylanase activity, the remaining supernatant was precipitated with 80% ammonium sulfate at 4°C for 2 h. After centrifugation at 25, 000 *g* for 40 min at 4°C, the protein precipitation was dissolved and dialyzed in 2 l PBS buffer (50 mM KH_2_PO_4_, K_2_HPO_4_, pH 6.2) for 6 h to remove ammonium sulfate. The solution after dialysis was used for SDS-PAGE. The cells harvested previously were washed with water and suspended in 1 ml of the extraction buffer containing 50 mM Hepes/NaOH, pH 7.0, 1 mM EDTA, 1 mM EGTA, 0.1 mM dithiothreitol, 2 µM *p*-amidinophenylmethanesulfonyl fluoride, and 300 mM sucrose. The cells were disrupted by vigorously vortexing with 1.5 g of glass beads (0.3 mm diameter) four times for 1 min at 4°C. The beads and the cell debris were removed by centrifugation at 20,000 *g* for 30 min. The cell extract and the extracellular samples were loaded onto 12% SDS-PAGE to examine the xylanase expression. The extracellular xylanase activity was determined in the culture supernatant by using the method described by Yin *et al.* [17].

### Stability assay of the plasmid pREP-AGCX

The cells of *S. pombe Δgfa1* were transformed with plasmid pREP-AGCX, and plated on YES agar medium. After grown for 2 days, the colony was streaked into YES liquid medium. The recombinant cells were cultured and transferred to fresh YES liquid medium at appropriate time to ensure the cells growing at logarithmic phase. After approximately 50 generations, the culture was diluted and spread on YES medium added with or without glucosamine, respectively. The number of viable colonies on each plate was then recorded.

## Results

### Construction and growth conditions of *E. coli Δglm*S

The *glm*S gene was deleted in *E. coli* K12 as described in Materials and Methods. On M9 minimal medium, *E. coli Δglm*S could grow only when glucosamine was supplemented, therefore, a defined medium could achieve the selective cultivation of the *E. coli Δglm*S cells transformed by using *glm*S as selection marker. However, in most circumstances, recombinant cells are cultivated in undefined media for fast growing, or are finally applied to nutritional rich environment, and thus an undefined medium has to be developed for the selective cultivation of GM bacteria with GFAT marker.

**Figure 3:**
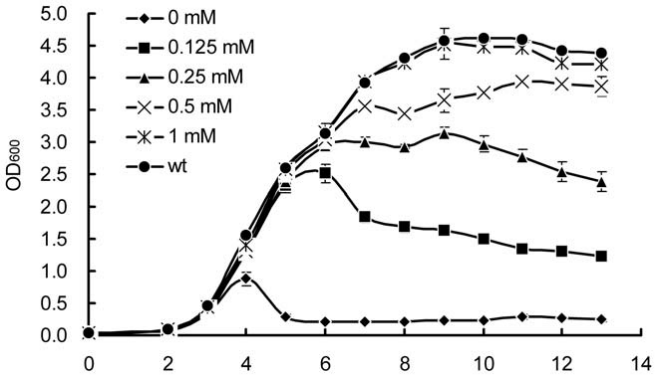
The growth condition of *E. coli* K12 *Δglm*S in LB medium supplemented with glucosamine at different concentrations.

*E. coli Δglm*S was able to grow on LB agar plates without addition of glucosamine, indicating that there was glucosamine in either yeast extract or tryptone. However, the glucosamine existed in LB broth could only support limited growth of *E. coli Δglm*S, the cell density declined rapidly after OD_600_ reached 0.8–1.0 (Fig. 3). The addition of glucosamine to LB medium increased the growth of *E. coli Δglm*S, and the growth rate of *E. coli Δglm*S was about the same to that of *E. coli* K12 when 1 mM glucosamine was supplemented (Fig. 3).

**Figure 4:**
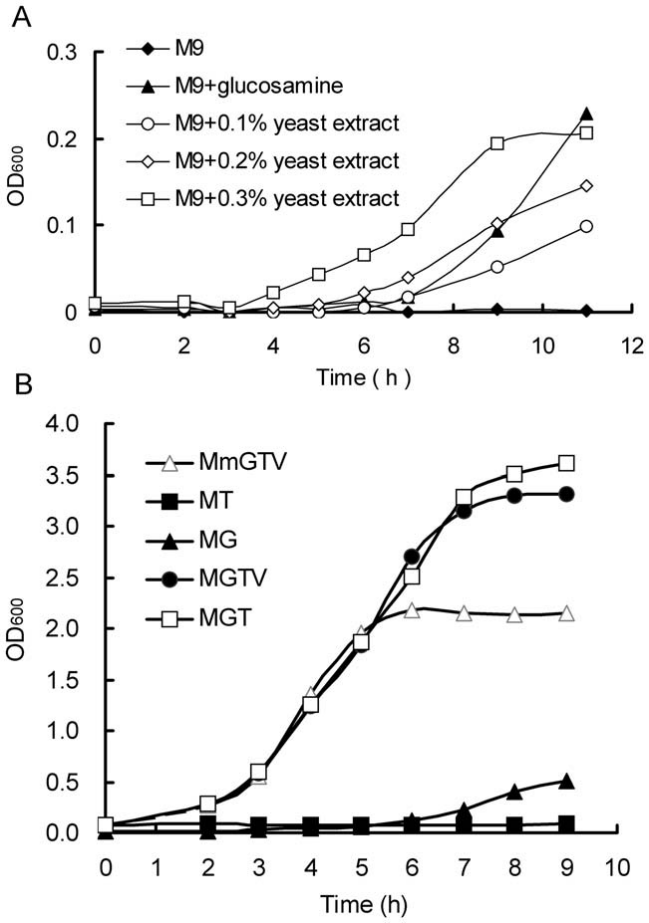
The growth condition of *E. coli* K12 *Δglm*S in different medium. (**A**) The growth condition of *E. coli* K12 *Δglm*S in M9 minimal medium supplemented with glucosamine and different concentration of yeast extract respectively. (**B**) The growth condition of *E. coli* K12 *Δglm*S in MmGTV, MT, MG, MGTV, MGT medium (M: M9 medium, Mm: M9 medium subtracting Mg^2+^ and glucose, G: glucosamine, T: tryptone, V: vitamin mixture).

It was found that cells of *E. coli Δglm*S could grow in M9 medium supplemented with yeast extract instead of glucosamine, in comparison, the cells grew slower in the medium containing 1 mM glucosamine than in that containing 0.3% yeast extract (Fig. 4A). The growth of *E. coli Δglm*S could result from the fact that the yeast extract contained amino acids and vitamins as well as glucosamine which would be exhausted in late growth phase at a low cell density (Fig. 4A).

No growth of *E. coli Δglm*S was observed in the M9 medium supplemented with 1% tryptone without the addition of glucosamine. Furthermore, the addition of tryptone in the M9 medium containing glucosamine greatly increased the growth of *E. coli Δglm*S, and thus overcame the shortage in poor nutrition of M9 medium. The addition of vitamins to the media did not increase the growth. In comparison, the M9 media (M) with glucosamine (G) and tryptone (T), MGT medium, was the best for *E. coli Δglm*S (Fig. 4B).

### As novel selection marker in a plasmid for *E. coli* system

The amp^R^ antibiotic gene was replaced with the *E. coli glm*S gene to give pHsh-glmS. This plasmid was transformed into *E. coli Δglm*S and selected on MT agar medium. Many colonies grew up on the selective plates and no colonies grew in the control. After isolation and enzyme digestion, plasmid pHsh-glmS was verified to be correct. The cells harboring pHsh-glmS could grow normally in the MT broth without glucosamine, and the SMG function of *glm*S in *E. coli* system was confirmed.

### Construction and growth properties of *S. pombe Δgfa1*

**Figure 5:**
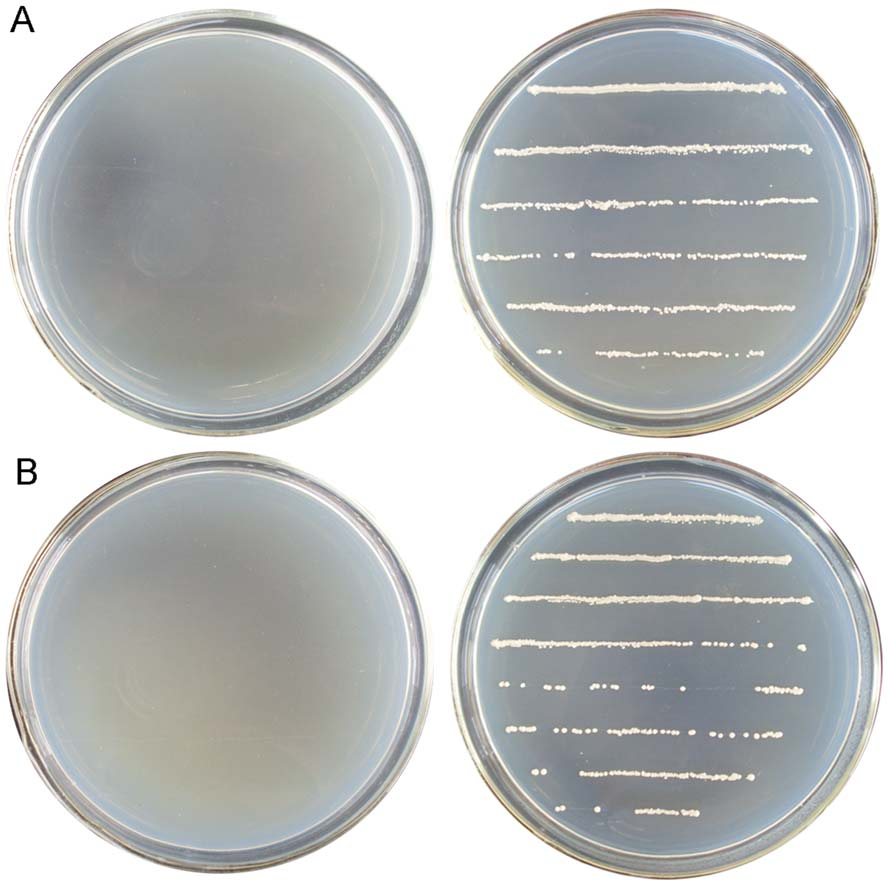
The growth condition of *S. pombe Δgfa1* on EMM and YES solid media with or without glucosamine. (**A**) *S. pombe Δgfa1* cells could not grow after streaking on EMM solid media without glucosamine (left), *S. pombe Δgfa1* cells grew well on EMM solid media supplemented with glucosamine (right). (**B**) *S. pombe Δgfa1* cells could not grow after streaking on YES solid media without glucosamine (left), *S. pombe Δgfa1* cells grew well on YES solid media supplemented with glucosamine (right).

**Figure 6:**
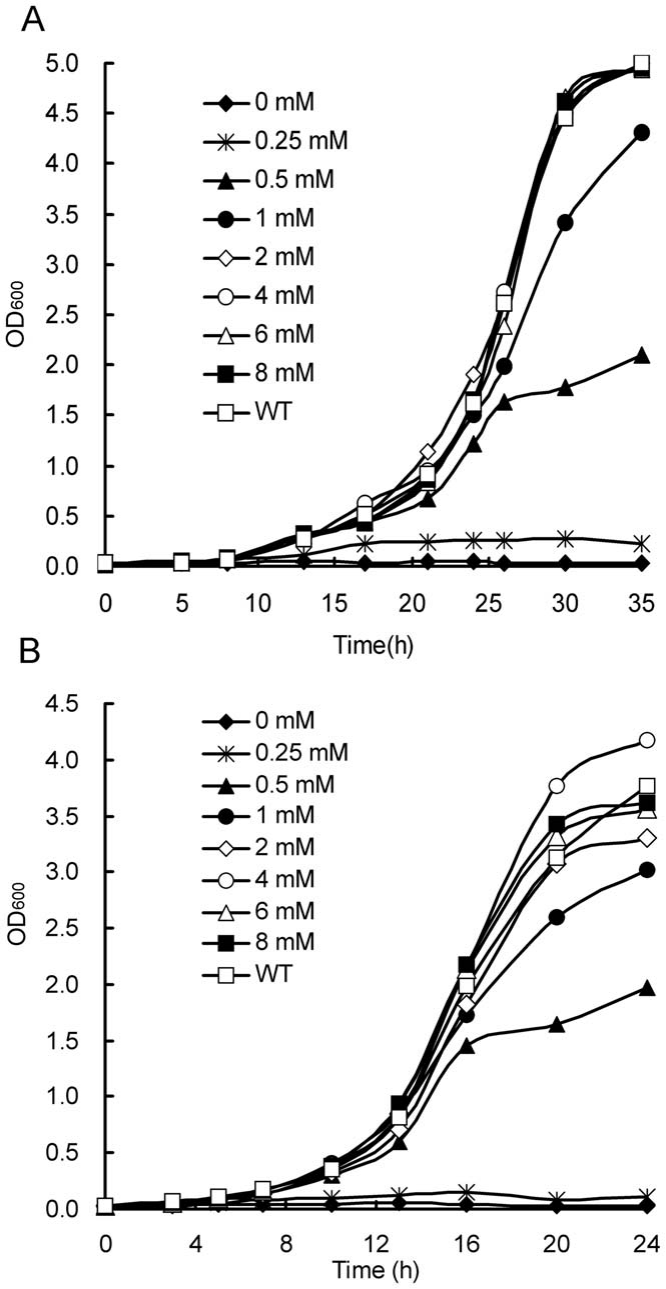
The growth condition of *S. pombe Δgfa1* in EMM and YES media supplemented with glucosamine at different concentrations. (**A**) *S. pombe Δgfa1* cells in EMM liquid media supplemented with glucosamine at 0, 0.25, 0.5, 1, 2, 4, 6, and 8 mM, respectively. (**B**) *S. pombe Δgfa1* cells in YES liquid media supplemented with glucosamine at 0, 0.25, 0.5, 1, 2, 4, 6, and 8 mM, respectively.

In order to assess the potential of GFAT as SMG in fungi, a *S. pombe* strain deleted for *gfa1* was constructed as described in Materials and Methods. The *S. pombe Δgfa1* strain could grow on glucosamine-supplemented YES and EMM solid media, but no growth was observed on the media without adding glucosamine (Fig. 5). In the EMM medium supplemented with 2 mM glucosamine, the *gfa1* deficient strain grew as fast as the host strain *S. pombe* YHL6381, further increase of glucosamine concentration was not beneficial to growth (Fig. 6A). The optimum concentrations of glucosamine supplemented to YES medium ranged from 2 to 4 mM (Fig. 6B).

### As novel selection marker in *S. pombe* plasmids

The expression plasmids pREP3X–gfa1 and pREP3X–gfat were obtained by cloning *S. pombe gfa1* or *V. volvacea gfat* into MCS of pREP3X, and then transformed into *S. pombe Δgfa1* cells. Colonies harboring pREP3X–gfa1 or pREP3X–gfat grew normally on EMM and YES medium without glucosamine after 2 days, while the cells transformed with pREP3Xdl did not grow on any of the media without glucosamine. This indicates that both GFAT genes from the host and the heterologous species, *V. volvacea*, can complement the *gfa1* deficiency of *S. pombe*.

### Heterologous gene expression mediated by *gfat* marker in *S. pombe Δgfa1*

**Figure 7:**
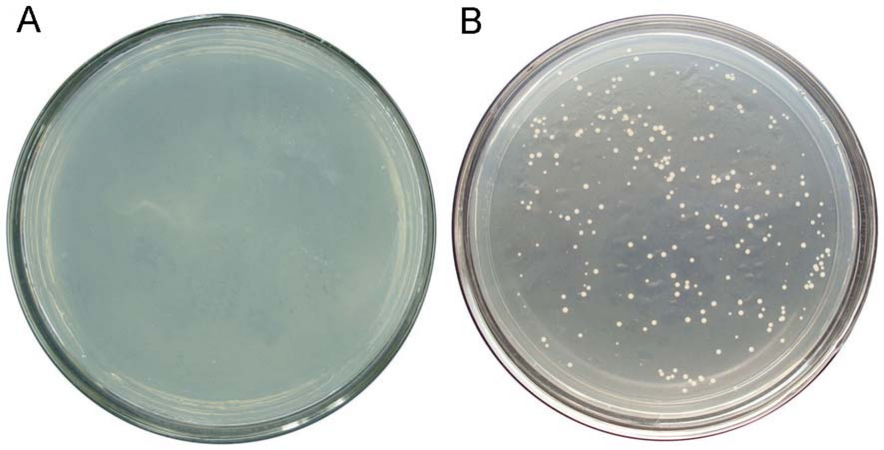
Transformation results of *S. pombe Δgfa1* with plasmids pREP-AGCX and pREP3Xdl-xynA. (**A**) Cells transformed with pREP3Xdl-xynA could not grow on the EMM medium without glucosamine. (**B**) Cells transformed with pREP-AGCX grew on the EMM medium without glucosamine.

**Figure 8:**
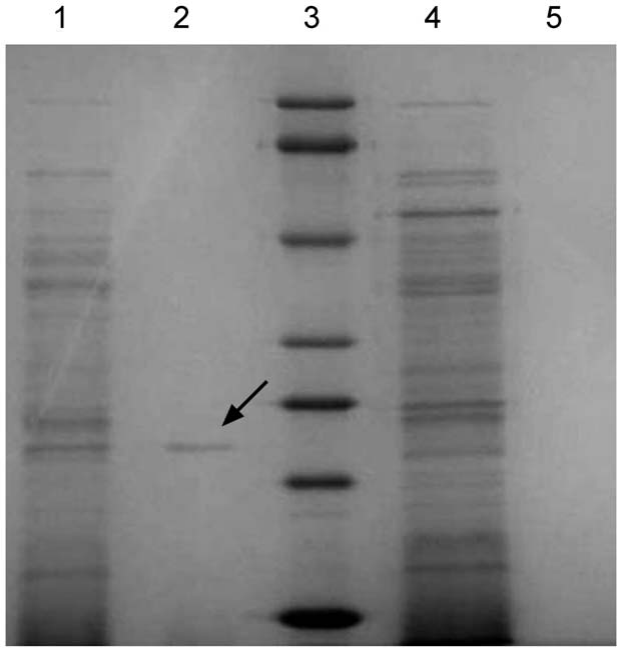
SDS-PAGE of the protein expressed from *xynA2* in *S. pombe Δgfa1*. Lanes: 1, crude extract of cells transformed with pREP-AGCX; 2, supernatant of cells transformed with pREP-AGCX; 3, molecular mass protein standards (top to bottom: 90, 66, 45, 34, 27, 20, 14.4 kDa); 4, crude extract of cells harboring pREP-AGX; 5, supernatant of cells harboring pREP-AGX. The gel was stained using Coomassie blue G-250. The amount of the protein loaded in lanes 1, 2, 4, 5 is 1.0, 0.2, 1.5, 0.4 µg, respectively. Lane 3 (protein standards): 0.5–1.0 µg of each protein.

The gene expression vector pREP-AG was successfully constructed by replacing the *LEU2* marker gene with *gfat* of *V. volvacea*. The plasmid pREP-AGCX was obtained after the xylanase gene fused with Cpy signal peptide was cloned into pREP-AG. The cells of *S. pombe Δgfa1* transformed with plasmid pREP-AGCX and pREP-AGX could grow on EMM medium without glucosamine, while cells transformed with control plasmid pREP3Xdl-xynA could not grow under this condition (Fig. 7). After *xynA2* was expressed in *S. pombe Δgfa1* cells transformed with pREP-AGCX at 32°C for 24 h, extracellular xylanase activity reached 28.8 U/ml. In the supernatant of the cells harboring pREP-AGX, no xylanase activity was detected. The molecular mass of the protein was 21 kDa on SDS-PAGE which was consistent with the original size (Fig. 8).

The stability of the plasmid selected by *gfat* marker was estimated through the growth of *S. pombe Δgfa1* cells carrying pREP-AGCX. After the cells were continuously grown for 50 generations, approximately 90% of the cells retained the plasmid pREP-AGCX during the tested time course. The high stability of the plasmid provided solid base for industrial application.

## Discussion

For the first time, the GFAT gene was proven to possess a function as an effective SMG for GM microbes, as suggested earlier [13]. This was demonstrated through the analysis of two GFAT deficient strains, *E. coli Δglm*S and *S. pombe Δgfa1*, and also the ability of the gene to mediate plasmid selection and stability.

The important role of GFAT gene was verified by the growth behavior of *E. coli Δglm*S and *S. pombe Δgfa1*. The importance of glucosamine in the medium was also established, both GFAT deficient strains failed to grow normally in some glucosamine minus media (G- medium). For example, YES G- medium did not support *S. pombe Δgfa1*, and similarly, M9 minimal medium did not support *E. coli Δglm*S, while LB G- medium allowed *E. coli Δglm*S to grow to a low cell density. Generally, tryptone can greatly improve the growth of *E. coli* in M9 minimal medium. However, there was no such impact on *E. coli Δglm*S when it was added to the medium. This confirmed that tryptone could be used for the gene selection on *E. coli* when using the *glm*S gene as an SMG. Therefore, MGT medium was almost as good as LB, as the optical density (OD) of *E. coli Δglm*S had reached a high level (Fig. 4B) after a few hours.

The concentration of glucosamine in the medium was shown to be fairly significant for both deletion strains. A very low dose of glucosamine (2–4 mM) in the EMM or YES medium could enable *S. pombe Δgfa1* to grow, the growth increased rapidly after a short delay (16 hrs). Its importance was illustrated further in *E. coli Δglm*S, it started to grow in M9 minimal medium when the yeast extract was added to it, which might contain glucosamine at a trace level. This suggested that the *Δglm*S strain was responding to a very low concentration of glucosamine in the yeast extract. This also demonstrated that *S. pombe Δgfa1* was less sensitive than *E. coli Δglm*S to glucosamine in some medium, possibly because yeasts need more glucosamine for the synthesis of chitin.

GFAT was successfully expressed in plasmids pREP3X-gfa1, pREP3X-gfat and pHsh-glmS in the transformants of *S. pombe Δgfa1* and *E. coli Δglm*S, and it complemented both GFAT gene deficient strains so that they could grow on the G- medium normally. The GFAT genes can be expressed under the constitutive promoters upstream on the expression vectors, which can compensate the deficiency of the cells. The GFAT gene-based host strains and plasmids offer a novel resource to perform gene transformation in microbes. As it is a natural component of most organisms, these strains could be used for GM food, pharmaceutical products as well as environmental remediation.

Furthermore, the GFAT gene was proved to be an effective SMG in the work, when the *xynA2* gene was successfully expressed and secreted extracellularly by plasmid pREP-AGCX in *S. pombe Δgfa1* for the expression product, xylanase, which possessed the same function as the original. This indicated that the GFAT gene could not only be used as a biosafe SMG, but also maintained its regulatory function in the metabolic pathway even if expressed by a heterologous eukaryotic organism.

In conclusion, the GFAT-encoding gene can be used in bacteria as a biosafe SMG instead of antibiotic resistant genes, it can also be used as a novel SMG for GM yeasts to be applied to complex media. These findings provide an effective technique for the construction of GM microbes for alternative purposes.
